# Hypothetical membrane mechanisms in essential tremor

**DOI:** 10.1186/1479-5876-6-68

**Published:** 2008-11-06

**Authors:** Aasef G Shaikh, Kenichiro Miura, Lance M Optican, Stefano Ramat, Robert M Tripp, David S Zee

**Affiliations:** 1 Department of Neurology, The Johns Hopkins University, Baltimore, MD, USA; 2 National Eye Institute, National Institutes of Health, Bethesda, MD, USA; 3 University of Pavia, Pavia, Italy; 4 FlexAble Systems, Fountain Hills, AZ, USA; 5 Graduate School of Medicine, Kyoto University, Kyoto, Japan

## Abstract

**Background:**

Essential tremor (ET) is the most common movement disorder and its pathophysiology is unknown. We hypothesize that increased membrane excitability in motor circuits has a key role in the pathogenesis of ET. Specifically, we propose that neural circuits controlling ballistic movements are inherently unstable due to their underlying reciprocal innervation. Such instability is enhanced by increased neural membrane excitability and the circuit begins to oscillate. These oscillations manifest as tremor.

**Methods:**

Postural limb tremor was recorded in 22 ET patients and then the phenotype was simulated with a conductance-based *neuromimetic *model of ballistic movements. The model neuron was Hodgkin-Huxley type with added hyperpolarization activated cation current (I_h_), low threshold calcium current (I_T_), and GABA and glycine mediated chloride currents. The neurons also featured the neurophysiological property of rebound excitation after release from sustained inhibition (post-inhibitory rebound). The model featured a reciprocally innervated circuit of neurons that project to agonist and antagonist muscle pairs.

**Results:**

Neural excitability was modulated by changing I_h _and/or I_T_. Increasing I_h _and/or I_T _further depolarized the membrane and thus increased excitability. The characteristics of the tremor from all ET patients were simulated when I_h _was increased to ~10× the range of physiological values. In contrast, increasing other membrane conductances, while keeping I_h _at a physiological value, did not simulate the tremor. Increases in I_h _and I_T _determined the frequency and amplitude of the simulated oscillations.

**Conclusion:**

These simulations support the hypothesis that increased membrane excitability in potentially unstable, reciprocally innervated circuits can produce oscillations that resemble ET. Neural excitability could be increased in a number of ways. In this study membrane excitability was increased by up-regulating I_h _and I_T_. This approach suggests new experimental and clinical ways to understand and treat common tremor disorders.

## Introduction

Essential tremor (ET) is a common neurological disorder characterized by postural tremor that worsens with movement. The pathophysiological mechanism of ET is unclear. However, a number of drugs that reduce neuronal membrane excitability are beneficial in ET. For example, propranolol – a commonly used beta-blocker that reduces membrane excitability [1] – is an effective drug for treating ET [2]. GABA-mimetic inhibitory agents such as gabapentin can reduce ET [3]. Ethanol, which enhances GABA and glycine mediated currents, and inhibits the excitatory glutamatergic transmission (ultimately decreasing neural excitability), ameliorates ET [4-6]. Improvement in ET was also reported with topiramate [7]. Topiramate has a GABA-mimetic effect and also inhibits glutamatergic (excitatory) transmission [8].

Therefore we hypothesize that increased membrane excitability in pre-motor neurons has a key role in pathogenesis of ET. First, we present two fundamental concepts that support this idea. Based upon these concepts, we then test our hypothesis with a conductance based computational model that simulates tremor.

### Concept 1 – Sherrington's 'burden': reciprocal inhibition and rebound depolarization

**Figure 1:**
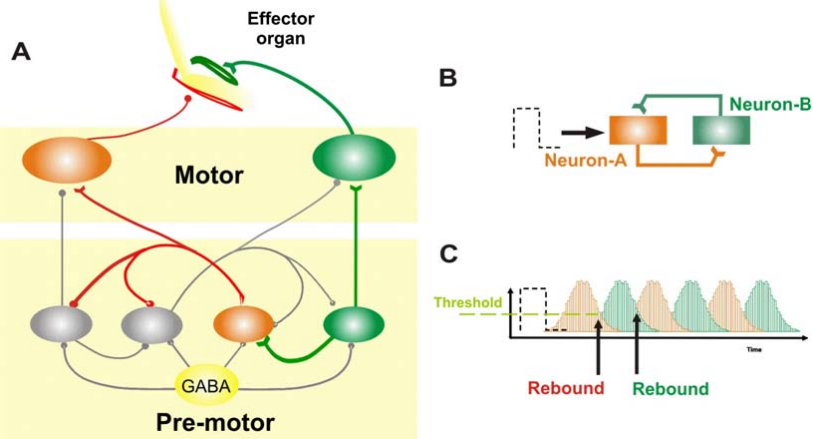
**(A) The circuit of reciprocally innervated neurons for controlling ballistic movements.** The excitatory premotor neurons send excitatory projections to the motor neurons innervating the agonist muscle group. At the same time this neuron also sends an excitatory projection to the inhibitory neuron innervating the motor neuron for the antagonist muscle group. In addition, mutual inhibitory connections exist between premotor neurons. These mutually inhibitory connections predispose the neural circuit to instability and oscillations. (B, C) Demonstration of oscillations in a two-neuron circuit. Neuron-A inhibits neuron-B and *vice-versa*. A small pulse to neuron-A increases its discharge and thus inhibits neuron-B. Once the discharge of the neuron drops inhibition from neuron-B is removed. This results in a rebound increase in the neuron-B firing rate. Since neuron-B also inhibits neuron-A, the same phenomenon of post-inhibitory rebound repeats for neuron-A. In the panel 'C' response of neuron A is schematized with red bars, while the green trace is response from neuron B.

Excitation of an agonist muscle and simultaneous inhibition of its antagonist is necessary for efficient force generation during movement. This is Sherrington's principle for reciprocal innervation. For example, when we flex an elbow, the flexor group of muscles receives excitatory impulses from the corresponding neurons. The same neurons also inhibit neurons innervating the antagonist muscle group, the extensors. Figure 1A schematically illustrates this phenomenon. The green lines are excitatory neural projections and red lines are inhibitory. Reciprocally inhibitory neural circuits are present in many central areas responsible for limb movement [9]. Furthermore, some limb movement related neurons also exhibit a rebound increase in their firing rate when inhibition is removed – *post-inhibitory rebound (PIR)*. For example, PIR is observed in neurons of premotor areas for limb movements, such as the thalamus and inferior olive [10].

Under physiological circumstances, with normal levels of external inhibition, PIR provides sufficient force to generate prompt, high-speed ballistic movements. Reciprocal innervation and PIR also make neural circuits inherently unstable and prone to oscillations [11,12]. Figure 1, panels B and C, illustrate why a circuit of reciprocally innervated neurons with PIR is prone to oscillations. A neural circuit is formed by two mutually inhibitory neurons (neuron-A and -B). A small pulse of neural activity, either from spontaneous fluctuations in neural activity or a small spontaneous movement, activates neuron-A. The discharge from neuron-A inhibits neuron-B. Following the termination of the input to neuron-A, its discharge drops and inhibition upon neuron-B is removed. Neuron-B in turn shows PIR and starts to fire. Since neuron-B also inhibits neuron-A, when firing in neuron-B stops, PIR occurs in neuron-A. Thus the reciprocally-innervated neural circuit begins to oscillate (Figure 1C).

Nature's solution to this inherent instability in a reciprocally innervated neural circuit is to add enough external inhibition to keep the excitability of the constituent neurons under control. For example, we have proposed that oscillations in an analogous circuit controlling ballistic eye movements (saccades) are normally prevented by tonic external glycinergic inhibition [12]. Similarly, abolishing GABAergic inhibition in GABA mutant mice is known to cause tremor [13]. However, oscillations in reciprocally innervating circuits can be seen in the presence of normal external inhibition. For example, in patients with ET GABA mutation was not found [14]. In the following section we introduce a concept explaining the basis of oscillations in reciprocally innervated circuits when external inhibition is intact.

### Concept 2 – Increased excitability can make reciprocally innervated neurons unstable

Oscillations in reciprocally innervated circuits could emerge if the relative effect of *intact *external inhibition is reduced by an increased excitability within the reciprocally-innervated neurons themselves. Therefore it is possible that increased neural excitability can overcome the effects of normal external inhibition. There could be a number of causes of increased excitability including an increase in either the hyperpolarization activated cation current (I_h_) or the low threshold calcium current (I_T_) [15,16] or alterations in the intracellular levels of second messengers and the regulators that influence the activation kinetics of these ion channels [16-18].

### Testing the hypothesis

We recorded postural limb tremor in 22 patients with ET, and used a neuromimetic model to simulate their tremor. We tested our hypothesis by simulating a Hodgkin-Huxley type, conductance-based, neuromimetic model of pre-motor burst neurons responsible for ballistic limb movements. This model has the following features: (1) A circuit consisting of reciprocally innervating excitatory and inhibitory neurons. (2) Physiologically-realistic membrane kinetics of the premotor neurons determined by specific and physiologically plausible subsets of membrane ion channels. The latter also determines the excitability of the membrane. (3) These model neurons had a property of *rebound firing *after sustained inhibition - post-inhibitory rebound (PIR). By increasing specific membrane conductances that are known to increase PIR and neural excitability, such as I_h _and I_T_, we could simulate the range of frequencies of tremor recorded from ET patients.

## Methods

### Patient selection and tremor recordings

We studied 22 ET patients, who gave written, informed consent before enrolling in the study.

#### Inclusion and exclusion criteria

Patients were recruited from the movement disorders clinic. Patients had bilateral postural tremor of their hands. We excluded patients with dystonia, drug-induced tremor, psychogenic tremor, and orthostatic tremor. Subjects with enhanced physiological tremor, which often resembles ET, were excluded. The frequency of the tremor and the effects of loading (putting weight on the outstretched limb) during recording of postural tremor condition were used to exclude patients with enhanced physiological tremor. Loading reduces the frequency of enhanced physiological tremor.

Limb tremor was recorded with a three-axis accelerometer attached with a piece of surgical tape to the top of the middle phalanx of the index finger (FlexAble Systems, Fountain Hills, AZ). Patients held their arms outstretched against gravity with palms toward the floor (postural tremor). Typical tremor frequency in ET does not exceed 15 Hz, thus we sampled at 100 Hz (more than three times the minimum Nyquist-Shannon sampling rate). The raw acceleration signal recorded by the three-axis accelerometer contains high-frequency noise, inherent in all acceleration sensing systems, and low-frequency noise due to changes in the attitude of the limb relative to gravity (sway). These artifacts were removed by de-trending and digital filtering that involved three-point averaging. The 1 G (9.8 m/s^2^) gravity vector, which is much larger than the tremor accelerations, was determined from the 3-D calibration and removed off-line [19].

Data from each axis of the accelerometer and the magnitude of the acceleration vector (square root of the sum of the acceleration squared on all three axes) were processed separately. Cycle-by-cycle analysis was performed. A cycle was defined as follows: first we removed any bias from the de-trended data (i.e., normalized amplitude = actual amplitude – mean amplitude). This kept the peaks of the cycles positive and the troughs negative. The time when the data trace changed from a negative value to a positive value (i.e., the positively moving zero-crossing) was recorded. The time of the first positively moving zero-crossing marks the beginning and the next positively moving zero-crossing marks the end of the given cycle. The inverse of the cycle period yields the cycle frequency; the difference between the peak and trough gives the peak-to-peak amplitude.

### Computational simulations

Here we summarize the key features of our computational model. Readers are referred to additional files 1, 2 and 3 for more details on methodology of computational simulations. The Hodgkin-Huxley equations were implemented to simulate action potentials. GABA and glycine mediated inhibitory chloride conductance was implemented for inhibition, and non-NMDA and NMDA glutamatergic channels for excitation. Kinematics of CaV3 channels (carrying I_T_) and four subtypes of HCN channel (HCN1-HCN4; carrying I_h_) were included to simulate PIR and to modulate neuronal excitability. The activation kinetics of each of the subtypes of ion channels carrying I_h _are different, HCN-1 being the fastest and HCN-4 the slowest, with the others having intermediate activation time constants [20].

The following equation describes the time evolution of the membrane potential of the brain stem neurons:

(1) C*dV/dt = -I_L _- I_T _- n1I_h1_- n2I_h2_- n3I_h3_- n4I_h4_- I_Na _- I_K _- I_Cl _- I_NMDA _- I_nonNMDA _

where V is the membrane potential of the neuron, C is the membrane capacitance (1 μF/cm2) and n1-n4 is a rate scaling factor determining the ion channel expression profile in the neuron. I_L_, I_T_, I_h1-4_, I_Na_, and I_K_, denote the leak current, low-threshold calcium current, hyperpolarization activated current (carried by HCN1-4), fast sodium current and delayed rectifier potassium current, respectively. I_Cl _is the synaptic current mediated by glycinergic and GABAergic neurotransmitters. I_NMDA _and I_nonNMDA _are synaptic currents mediated by NMDA and non-NMDA sensitive glutamate receptors. The parameters n1-n4 represent the relative expression strength of I_h _channels. The model simulated a set of four burst neurons – inhibitory agonist, inhibitory antagonist, excitatory agonist, excitatory antagonist. We assumed a small, yet physiologically plausible, variability in the ion channel expression profiles of the simulated burst neurons, which in turn, accounted for minor (physiological) variability in their membrane properties. Supplementary figure 1 (additional file 2 ) schematizes the model organization, synaptic weights, and mathematical functions implemented in the model. Additional details of the model are in additional file 1  as well as in Miura and Optican (2006) and Shaikh, et al., (2007).

## Results

### Model simulations

**Figure 2:**
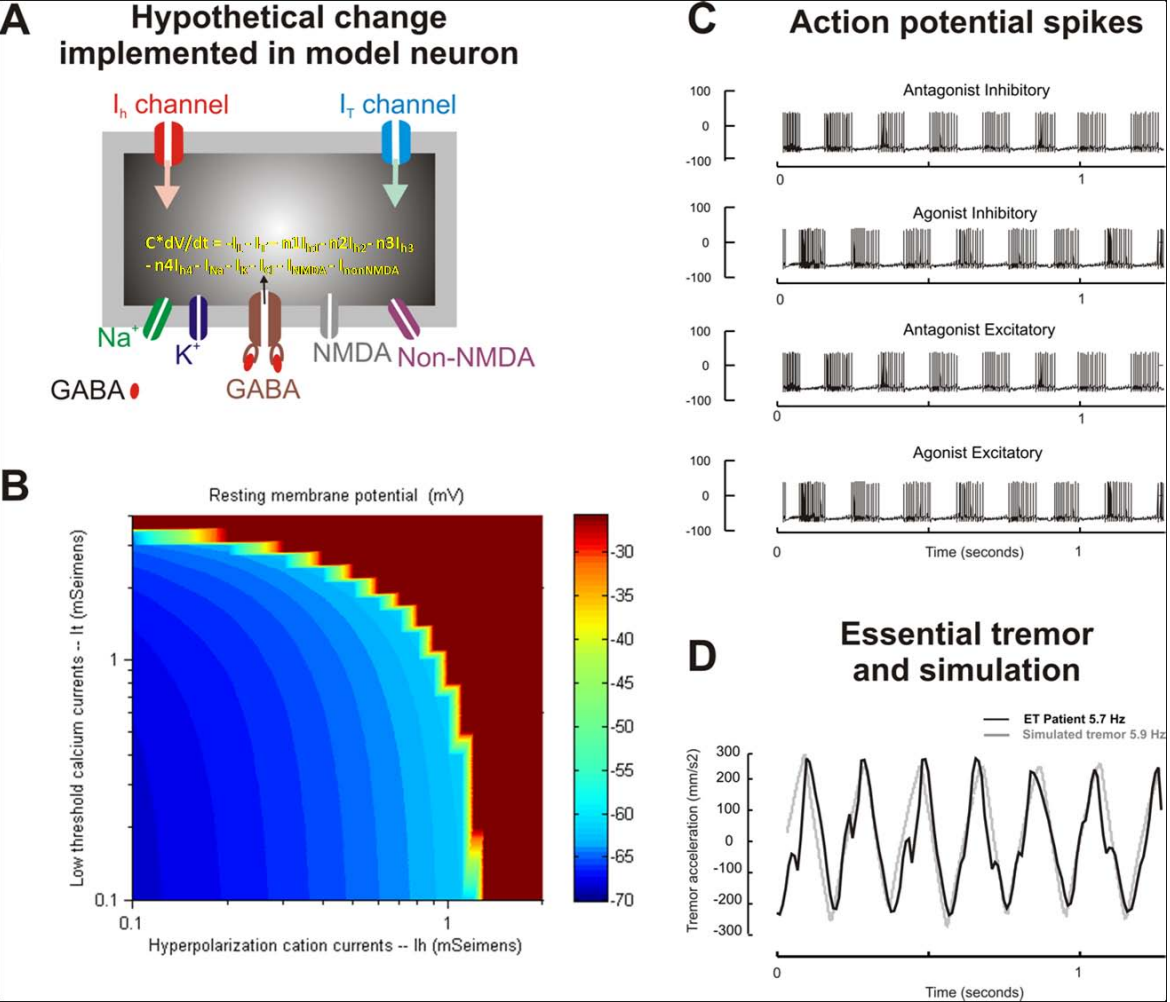
**(A) A traditional Hodgkin-Huxley model of cell membranes with multiple ion channels was used to generate the action potential.** In order to simulate physiologically realistic neural behavior, ion channels such as hyperpolarization activated cation currents (I_h_) and low threshold calcium current (I_T_) were also included. NMDA and non-NMDA excitatory glutamatergic channels as well as GABA sensitive inhibitory channels were also included. The grey box schematizes the burst neuron, while its grey outline schematizes the cell membrane. The ion channels span the membrane thickness. dV is the rate of change in the membrane potential over period 'dt'. C is the membrane capacitance (1 μF/cm2) and n1-n4 is a rate scaling factor determining the ion channel expression profile in the neuron. I_L_, I_T_, I_h1-4_, I_Na_, and I_K_, denote the leak current, low-threshold calcium current, hyperpolarization activated current (carried by HCN1-4), fast sodium current and delayed rectifier potassium current, respectively. I_Cl _is the synaptic current mediated by glycinergic and GABAergic neurotransmitters. I_NMDA _and I_nonNMDA _are synaptic currents mediated by NMDA and non-NMDA sensitive glutamate receptors. (B) The effects of changing I_h _(x-axis) and I_T _(y-axis) on the resting membrane potential (color coded) in the simulated neuron. As expected, increases in I_h _and I_T _further depolarize the neuron. A depolarizing shift in the resting membrane potential reflects increased neural excitability. (C) Illustration of bursts of action potential spikes from the agonist and antagonist burst neurons. The alternate spiking behavior of these neurons is evident when they are plotted along the same time scale (x-axis). (D) Simulation (grey trace) of essential tremor (black trace) is shown. The tremor amplitude (y-axis) is plotted against time (x-axis). The time scale for simulated essential tremor is the same as the time scale for the traces representing the spiking behavior of the burst neurons. The frequency of tremor recorded from the patient is 5.7 Hz, which is closely simulated by the neuromimetic model (5.9 Hz). The amplitude of the simulated tremor also resembles the one recorded from the ET patient.

We simulated membrane properties of reciprocally-innervated burst neurons within a local feedback loop model for ballistic movements. Each neuron was a conductance-based single-compartment model. The major conductances simulating the membrane properties of the model neurons are schematized in Figure 2A. The details of the model organization and the mathematical equations driving these ion currents are explained in methods and additional file 1, 2 and 3. The model is compatible with the known anatomical organization of neural circuits for limb movements. The membrane properties of the model burst neurons are also consistent with the known profiles of limb-movement sensitive pre-motor neurons. The model simulated normal ballistic limb movements when the ion currents and excitability of the simulated burst neuron membranes were within physiological limits (I_h _= 0.1 mSeimens; I_T _= 2.0 mSeimens; and resting membrane potential = -68 mV). Increases in I_h _and I_T _further depolarized the resting membrane potential producing increased neural excitability (Figure 2B). Oscillations resembling ET were simulated when I_h _and I_T _in the model neurons were increased. The increase in these currents resulted in alternating bursts of action potentials in the neurons innervating the sets of agonist and antagonist muscles (Figure 2C). In Figure 2C the membrane potential is plotted along the y-axis and time along the x-axis. The alternating bursts of discharge reflect the circuit oscillations and produce the limb tremor. The simulated oscillations (the model output) are illustrated as the grey trace in Figure 2D. The common time scale in Figure 2C and 2D facilitates the comparison of the simulated oscillations (model output) with the alternating bursts of discharge in the pairs of neurons innervating agonist and antagonist muscles. The black trace in Figure 2D illustrates a representative postural limb tremor recorded from one ET patient. The simulated tremor superimposes fairly well on the postural limb tremor from the ET patient. The frequency of the simulated tremor (5.9 Hz) is close to the actual tremor frequency (5.7 Hz). The correlation coefficient between the two waveforms was 0.9.

**Figure 3:**
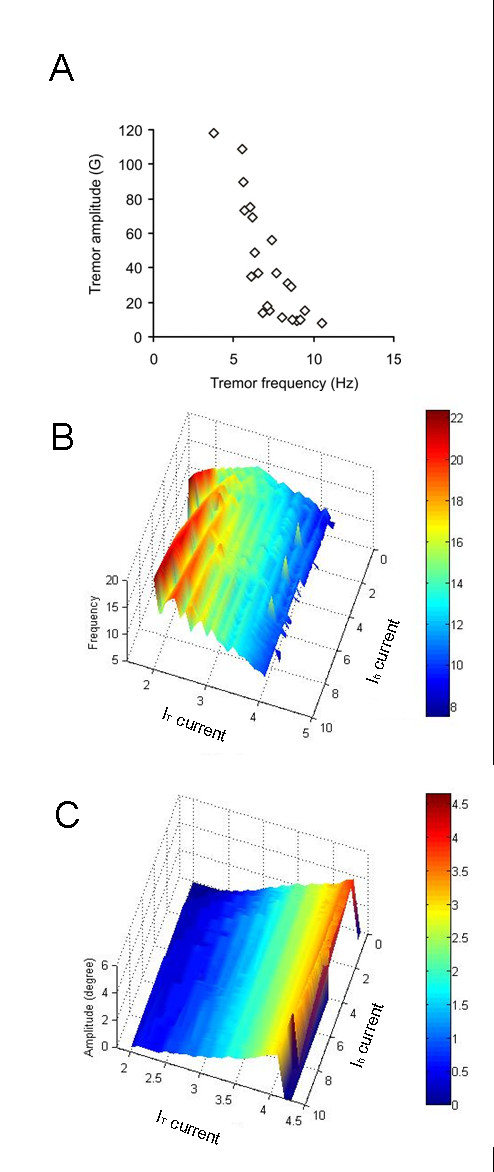
**(A) Correlation of frequency and amplitude of tremor in 22 ET patients.** Each data point represents one patient. A negative correlation was noted between the frequency and amplitude in 22 ET patients. Also note the frequency of ET ranges from 3–11 Hz in the patients. G = 0.0098 m/s^2^. (B) The frequency of the oscillations is determined primarily by I_h _and I_T_, which in turn depend upon the distribution of ion channel subtypes (upper panels). The frequency of oscillation is predominantly determined by I_h_, while its amplitude is predominantly determined by I_T_. Notice in panel 'B' that as we increase I_T _the red color appears sooner for higher values of I_h _but is not present when I_h _is relatively low (~2 ms). (C) The amplitude of tremor is predominantly determined by the value of I_T_.

Postural tremor in ET has a relatively wide range of frequencies [21]. Therefore, we asked if the conductance-based membrane model could simulate the inter-subject variability in the tremor frequency. The range of frequencies and corresponding amplitude of the postural tremor from 22 ET patients are illustrated in Figure 3A. The tremor frequency in these ET patients ranged from 3–11 Hz. We simulated this variability in frequency by changing the value of I_h _and I_T_. How the amplitude and frequency of tremor depend on the value of I_h _and I_T _in our model is shown in Figure 3B and 3C. In these figures the x- and y-axes represent I_T _and I_h_, respectively. The oscillation frequency (Hz) and amplitude (degree) are color coded and plotted along the z-axes. Tremor occurred when conductance through I_h _was larger than 1 mS/cm^2 ^(approximately 10 times larger than its physiological value, 0.1 mS/cm^2^; S: seimens) [22,23]. The effects of increase in I_h _on changes in frequency and amplitude, for a given constant value of I_T_, were investigated. Increasing I_h_, while keeping I_T _at a constant value, increased the frequency of tremor (slope ± 95% confidence interval: 0.5 ± 0.05). However, when I_T _was kept constant, there was only a slight decrease in the tremor amplitude when I_h _was increased (slope ± 95% confidence interval: -0.02 ± 0.10). Then we investigated the changes in the frequency and amplitude of tremor when I_T _was increased but I_h _was kept at a constant value. With a constant I_h_, the tremor frequency decreased when I_T _was increased (slope ± 95% confidence interval: -3.9 ± 0.1). In a similar analysis, the amplitude of tremor, however, increased with increasing I_T _(slope ± 95% confidence interval: 1.1 ± 0.01).

If I_h _was increased above the physiological values, ET was simulated for any I_T _that was 2.75 mS/cm^2 ^or larger (normal I_T_: 2.0 mS/cm^2^, reference 26). In contrast, if I_h _was near its physiological range, increasing I_T _alone did not simulate oscillations.

**Figure 4:**
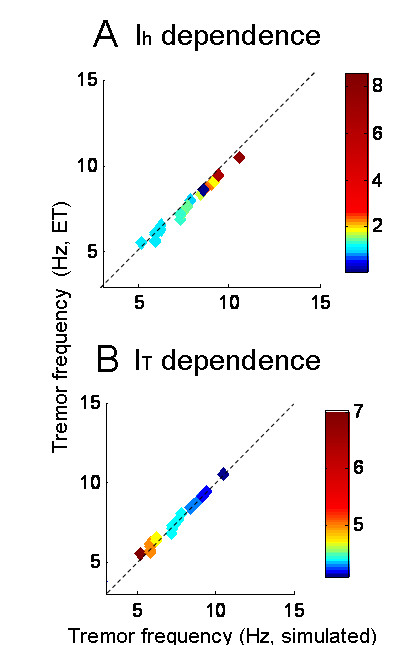
**Strong correlation between the frequency of simulated tremor and corresponding postural tremor frequency in the ET patients.** Note that all the data points fall along the equality line suggesting a strong correlation. The color coding illustrates the value of I_T _or I_h _in the model that was required to simulate tremor from each ET patient. (A) The higher values of I_h _correspond to higher amplitude as well as frequency of tremor. The range of modeled I_h _conductances to simulate the ET is 10 – 80 times larger than its physiological value. (B) The higher values of I_T _in the model corresponds to the lower frequency. In contrast to I_h_, the range of I_T _to simulate ET is only 1.3 – 3 times higher than its physiological value. These results imply that tremor frequency amongst ET is predominantly determined by the I_h _(as compared with I_T_).

We tuned the model to match the tremor frequency measured in each of the 22 ET patients. Then we plotted the observed versus simulated frequency for each patient as colored diamonds, indicating the model values of I_h _and I_T _required for that patient (Figure 4). There was a nearly perfect correlation between the frequency of simulated tremor and the corresponding postural tremor in the patients (all the data points fall along the black dashed equality line). The data points in Figure 4 are color-coded according to the value I_T _or I_h_. The higher values of I_h _correspond to ET patients with higher tremor frequencies (Figure 4A). Consistent with the results in Figure 3, the range of I_h _to simulate ET was 10 – 80 times larger than its normal physiological value. The corresponding range of I_T _to simulate the characteristics of ET was only 1.3 – 3 times higher than its physiological value. Our results suggest that the variability of tremor frequency among ET patients is determined by both I_h _and I_T_. Tremor frequency increases with increasing I_h _and decreases with increasing I_T_.

In summary, simulations support our hypothesis that an increase in premotor neural excitability, caused by increasing I_h _and I_T_, results in oscillations of a reciprocally innervated neural circuit. These oscillations resemble ET. The frequency of tremor is determined by both I_T _and I_h_.

## Discussion

Essential tremor (ET) is a common but poorly understood neurological disorder [25,26]. Patients with ET, however, are reasonably well treated with drugs that reduce membrane excitability. Therefore, we asked whether or not increased membrane excitability could play a critical role in the pathogenesis of ET.

### How increased membrane excitability affects motor control?

Increases in membrane excitability could affect motor control in many ways. One mechanism is by destabilizing the circuits comprised of reciprocally innervated neurons. Such circuits exist at many levels in the central nervous system. This pattern of reciprocal innervation between pre-motor neurons projecting to a pair of agonist-antagonist muscles is fundamental for efficient force generation during ballistic movements [9]. The stability in these circuits requires adequate external inhibition. Either removal of external inhibition [12] or increasing the excitability of the constituent neurons (as proposed here) could lead to oscillations that produce tremor.

### Can hyperexcitability cause oscillations in motor circuits that lead to tremor?

We used a conductance-based model of burst neurons with physiologically realistic membrane properties and anatomically realistic neural connections to test this hypothesis. The cardinal features of this model were threefold: 1) increased neural excitability secondary to increase in I_h _and/or I_T_, 2) post-inhibitory rebound (PIR), and 3) inherent circuit instability resulting from reciprocal innervation between the neurons projecting to agonist and antagonist muscle pairs. The model simulated oscillations resembling ET when I_h _was increased (with or without an increase in I_T_). While suggesting an overall conceptual framework underlying oscillatory behavior, our model can not pinpoint the specific anatomical neural networks that produce ET. Nevertheless, we can reasonably suggest that the following anatomical regions might be involved.

### Neural circuits that might oscillate

Circuits in the thalamus, inferior olive, cerebrum and cerebellum are involved in the generation of limb movements. One mechanism for tremor is that a group of neurons within a single nucleus develops an abnormal oscillatory mode. In this mode, a neural discharge is followed by a prolonged hyperpolarization that terminates in rebound spikes. Thus, each neuron oscillates independently. Synchronization of such independently oscillating neurons could result in rhythmic activity that becomes strong enough to cause gross motor oscillations. Electrotonic coupling through connexin gap junctions can facilitate synchronization in premotor nuclei such as the inferior olive [27]. Cells of the inferior olive also express ion channels that carry I_h _and I_T_. Moreover, I_h _is thought to influence the synchronization of oscillations in the inferior olive [28]. It is possible that increased intracellular levels of cAMP in the inferior olive increases I_h _conductance to facilitate these oscillations. Octanol, which reduces synchronized oscillations in the inferior olive, also reduces ET [29,30].

Reciprocal innervation occurs in many neural circuits controlling limb movements [9,12]. For example, thalamo-cortical relay (TC) neurons send glutamatergic excitatory projections to thalamic reticular (TR) neurons. TR neurons send GABA mediated inhibitory projections to TC neurons [31-33]. TR neurons mutually inhibit each other via inhibitory collaterals [31-33]. Thus interactions between TC and TR neurons make reciprocal loops with positive feedback from the TC to TR neurons, negative feedback from TR to TC neurons, and mutually inhibitory TR neurons [34]. The globus pallidus internus (GPi) sends inhibitory projections to TC and TR neurons in the motor thalamus [35,36]. Furthermore, thalamic and GPi neurons carry I_h _and I_T_, and exhibit post-inhibitory rebound [1,15,37,38]. Hence, the reciprocally innervated neural network formed by the TC and TR neurons might be inherently unstable [12]. Physiologically, this network is under control by external inhibition from the GPi.

Bursting behavior in neurons, which is fundamental for making a circuit prone to oscillations, is also seen in the subthalamic nucleus [39]. Reciprocal innervation – a key feature for oscillating neural circuits – is also apparent in the spinal cord [9]. Thus there are a number of areas related to motor control within the central nervous system that are comprised of neurons and circuits that are prone to oscillations.

### Increasing the membrane excitability in our lumped model

In our model we increased the I_h _to increase membrane excitability and produce oscillations. As this is a lumped model, we can not differentiate among increasing the maximal conductance of an individual channel, increasing the number of channels, or increasing the probability that a channel is open. Any combination of these changes would increase I_h_. Likewise, there are a number of intracellular modulators regulating I_h _including intracellular levels of calcium, cAMP, and pH [15,16] that could affect membrane excitability. Again, our simulated lumped model cannot tell us which of these factors might be the cause. We had to increase I_h _by ten-fold to simulate tremor. Although the range of pathological changes in channel currents is not known, a ten-fold increase does not seem unreasonable, as experimental studies have shown that conductances can be changed several fold [17]. Thus, the conceptual underpinning of our lumped model seem plausible.

Naturally, we do not expect that all patients with ET have the same cause for their increased excitability and consequent tremor. But, with the wide range of ion channels that participate in the molecular cascade that accounts for PIR, many types of alterations could change membrane excitability and in turn lead to the phenotype of ET. Similarly, the effects of loss of inhibition from the cerebellar Purkinje neurons (which may be abnormal in some patients with ET, see below) might also change the membrane threshold and increase the excitability of the premotor neurons, and lead to the appearance of tremor in some ET patients [25,26]. Indeed, our model underscores the importance of external inhibition in preventing oscillations in a reciprocally innervated circuit. For example, removal of external inhibition in an analogous model accounted for limb tremor in a syndrome called micro-saccadic oscillation and limb tremor – microSOLT [12].

Some ET patients have the gly9 susceptibility variant of the DRD3 gene [40,41]. DRD3 receptor is expressed in the thalamus and substantia nigra where limb-movement related neurons are present [42]. Two of the possible effects of this mutation could be directly related to the regulation of membrane excitability. There is a prolonged intracellular action of mitogen-activated protein kinase (MAPK) in the gly9 variant of DRD3 [40]. The latter can cause increased intracellular levels of cAMP via excessive inhibition of phosphodiesterase E4 [43-45]. Conversely, it was also shown that gly9 variant in DRD3 gene is associated with reduced forskolin induced formation of cAMP [40]. Although it is not known what the intracellular levels of cAMP in ET are, it is possible that they are altered. If they are higher, I_h _could be increased and thus lead to an increase in membrane excitability.

### Co-existing changes in central nervous system to change oscillation kinematics

Our simulations suggested that increasing I_h_, and thus increasing excitability, increases the tremor frequency. It was also reported that the frequency of ET decreases and the amplitude increases with age [46]. Aging might alter the profile of membrane ion channels; our model explains the effects of maximal ion conductance on the tremor frequency. Furthermore, our simulations suggest that a parallel increase in I_T_, when I_h _is already increased, reduces the frequency of the tremor (Figure 3B) and increases its amplitude (Figure 3C). This explanation does not exclude the possibility that aging could also change other membrane and circuit properties including the latency of the long feedback loop around the burst neurons.

### Caveats and future directions

Although our hypotheses remain to be proven experimentally, they suggest new approaches to understanding common tremor disorders. The conceptual scheme that we present here could also be used to analyze tremor disorders other than ET. However, the unique aspect of this model is that it can simulate oscillations by changing intrinsic membrane properties of the burst neurons and does not require any 'structural' changes in the anatomical organization or connectivity of the constituent neurons. The latter is relevant to ET, since there are no gross structural changes except in some cases in which there is a decrease in cerebellar Purkinje neurons [25,26]. Loss of external inhibition to reciprocally innervating circuits could be an important underlying pathophysiological mechanism component in other tremor disorders including Parkinson's disease, cerebellar tremor, and micro-saccadic oscillations and limb tremor [12,34].

Finally, treatment with pharmacological blockers targeted towards ion channels may offer therapeutic benefits. For example, although counterintuitive, *interfering *with the function of a normal ion channel to decrease membrane excitability in the face of impaired external inhibition might reduce oscillatory behavior. Such an approach is similar to that for some forms of inherited epilepsy in which seizures are presumably caused by an abnormal ion channel. Treatment then can target another, presumably intact channel [47].

## Competing interests

The authors declare that they have no competing interests.

## Authors' contributions

AS formulated the hypothesis and experimental design, carried out the experimental studies, participated in the model development, analyzed data, and wrote the manuscript. KM developed the model. LO participated in the model development and manuscript writing. SR participated in model development. RT collected the experimental data and prepared the experimental data analysis software. DZ formulated the hypothesis, experimental and modeling design, and wrote the manuscript. All authors read, edited, and approved the final manuscript.

## Supplementary Material

**Additional file 1** **Supplementary material** Click here for file

**Additional file 2** Membrane based model for essential based tremor Click here for file

**Additional file 3.  Voltage dependences of H-current** Click here for file
