# Supplementary material for: Hypothetical membrane mechanisms in essential tremor
Source: J Transl Med. 2008 Nov 6;6:68. doi: 10.1186/1479-5876-6-68 (PMC2613385; doi:10.1186/1479-5876-6-68)
Supplement: Additional file 1 — Supplementary material [file 1479-5876-6-68-S1.doc]

**Supplementary material**

We used a conductance-based, single-compartment model of burst neurons to examine the contributions of the biophysical properties of membrane channels and membrane excitability to generate oscillations. We started with the Hodgkin-Huxley equations for modeling the action potential. Five other channels were added to model the effects of excitatory and inhibitory inputs. A hyperpolarization activated cation current (I_h_), low threshold calcium current (I_T_) were added to model the post-inhibitory rebound (PIR), a GABA and glycine mediated chloride conductance, and non-NMDA and NMDA channels were used to model excitatory inputs. A functionally analogous model was also simulated to study saccadic oscillations and saccadic eye movements (Miura and Optican, 2006). Some of the details of burst neurons used in this model are similar to that used in Miura and Optican (2006), however, adding I_h_ and GABA mediated chloride conductance (I_Cl_) were significant enhancements of the model used in Miura and Optican (2006). Furthermore, the current model has anatomical connections (neural circuit), membrane parameters and ion channel profiles that are better suited to limb movement sensitive pre-motor areas of the central nervous system.

**The conductance-based membrane model for burst neurons**

The equation used for describing the time evolution of the membrane potential of a neuron was:

C*dV/dt = -I_L_ - I_T_ – n1I_h1_- n2I_h2_- n3I_h3_- n4I_h4_ - I_Na_ - I_K_ - I_Cl_ - I_NMDA_ - I_nonNMDA_

where dV/dt is the rate of change in the membrane potential of the burst neuron, C is the membrane capacitance (set to 1 μF/cm^2^). I_L_, I_T_, I_h_, I_Na_,I_Cl_, and I_K_, denote the leak current, low-threshold calcium current, hyperpolarization activated cation current, fast sodium current, GABA and glycine activated chloride current, and delayed rectifier potassium current, respectively. I_NMDA_ and I_non-NMDA_ are synaptic currents mediated by glutamatergic synapses. (Note that inward currents have a positive sign.)

**Leak current**

The leak current was modeled as I_L_=g_L_(V-E_L_) where V is membrane potential, g_L_= 0.4 mS/cm^2^ and E_L_ = −70 mV represent the conductance and reversal potential of the leak current, respectively.

**Hyperpolarization activated cation current (I_h_)**

We adopted the I_h_ model that was originally described by Huguenard and McCormick (1992). The I_h_ is described as follows:


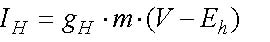

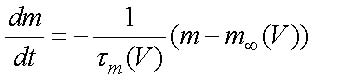


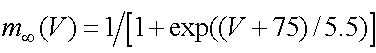


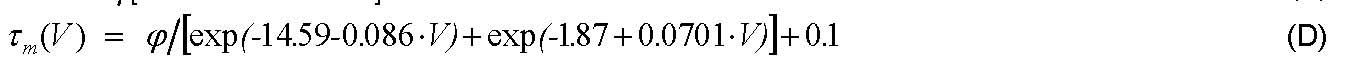


where, g_H_ and E_H_ (-40 mV) represent the maximal conductance and the reversal potential of this channel, respectively. The activation variable is m, and m_∞_ and τ_m_ are its steady state value and its time constant, respectively. φ is the scaling constant for the time constant of the activation variable. The properties of this channel are summarized in supplementary Figure 2 A, B.

**Low threshold calcium current (I_T_)**

I_T_ simulation was adopted from the model developed by Destexhe, et al., (1994). Equations for the dynamics of I_T_ current are as follows:

In these equations, g_T_ represent maximal conductance while E_T_ (= 120 mV) is the reversal potential. The activation variable is m, and m_∞_ and τ_m_ are its steady state value and its time constant, respectively. The inactivation variable is h, and h_∞_ and τ_h_ are its steady state value and its time constant, respectively. Detailed properties of this channel are explained in Miura and Optican (2006).

**Fast sodium and potassium currents**

The action potentials were modeled using Hodgkin-Huxley kinetics for the sodium current (I_Na_) and the delayed rectifier current (I_K_). The burst neurons can produce a train of action potentials with unusually high frequency, i.e. over 1 kHz, during saccades. Here, to simulate action potentials of the burst neurons during ballistic movements, we scaled the kinetics of the Hodgkin-Huxley model according to the modifications of Enderle and Engelken ([1995](http://www.springerlink.com/media/chmntjvqypuhe1lp8fvk/contributions/y/w/w/2/yww25342p87l7752_html/fulltext.html#CR10%23CR10)). In addition, we chose a recent formalism for the kinetics of I_Na_ and I_K_ used in a model of hippocampal pyramidal cells by Traub et al. ([1991](http://www.springerlink.com/media/chmntjvqypuhe1lp8fvk/contributions/y/w/w/2/yww25342p87l7752_html/fulltext.html#CR57%23CR57)). The equations for I_Na_ are as follows:

where *g_Na_* (= 120 mS/cm^2^), *E_Na_* (= 45 mV) and ᵠ denote the maximal conductance, the reversal potential of this channel and a scaling constant, respectively; m and h are, respectively, activation and inactivation variables of the sodium channel. α_m_(V) and β_m_(V) are the voltage-dependent forward and backward rate variables for activation, and α_h_(V) and β_h_(V) are those for inactivation. The equations for I_K_ are:

where g_K_ (=10 mS/cm^2^) and E_K_ (= −95 mV) denote the maximal conductance and the reversal potential of this channel, respectively, and n is the activation variable of the potassium channel. α_n_(V) and β_n_(V) are the voltage-dependent forward and backward rate variables for activation. To fit the burst neuron firing rate, the kinetics of the sodium and potassium currents are multiplied by 8 (i.e., ᵠ was set to 8), so that the model can generate action potentials at over 1 kHz. This implementation was necessary to examine the behaviors of the I_T_ during the high frequency oscillation of membrane potential during ballistic limb movement.

**Glutamatergic currents**

The predominant fast excitatory neurotransmitter of the vertebrate central nervous system is glutamate (Koch, [1999](http://www.springerlink.com/media/chmntjvqypuhe1lp8fvk/contributions/y/w/w/2/yww25342p87l7752_html/fulltext.html#CR28%23CR28)). There are two major subclasses of glutamate channel receptors, a rapid type (non-NMDA receptors, e.g., AMPA receptors) and a slower type (NMDA receptors), which usually co-exist (McBain and Mayer, [1994](http://www.springerlink.com/media/chmntjvqypuhe1lp8fvk/contributions/y/w/w/2/yww25342p87l7752_html/fulltext.html#CR33%23CR33)). We incorporated both channel types in our model, hence I_glu_ is defined as I_nonNMDA_+I_NMDA_. The kinetics of I_nonNMDA_ were essentially taken from the model of the AMPA receptor in Tegnér et al. ( [2002](http://www.springerlink.com/media/chmntjvqypuhe1lp8fvk/contributions/y/w/w/2/yww25342p87l7752_html/fulltext.html#CR55%23CR55) ).

where g_nonNMDA_, E_nonNMDA_ (=0 mV) and s denote the maximal conductance, the reversal potential and the opening probability of this channel, respectively; α_nonNMDA_ and τ_nonNMDA_ (= 2 ms, which is taken from Tegnér et al. ([2002](http://www.springerlink.com/media/chmntjvqypuhe1lp8fvk/contributions/y/w/w/2/yww25342p87l7752_html/fulltext.html#CR55%23CR55))) are the activation rate and time constant of deactivation, Glu_in_ is the drive input to the MLBNs from upstream, which is related to the motor error (in degrees, for details see below). The activation rate, α_nonNMDA_, was set to 0.1 ms^−1^deg^−1^. The kinetics of I_NMDA_ were modified from Tegnér et al. ([2002](http://www.springerlink.com/media/chmntjvqypuhe1lp8fvk/contributions/y/w/w/2/yww25342p87l7752_html/fulltext.html#CR55%23CR55)) with an added term describing the effect of glycine concentration around the receptor. The equations are as follows:

where g_NMDA_, E_NMDA_ (=0 mV), s and s_0_ denote the maximal conductance, the reversal potential, the channel opening probability and a synaptic variable proportional to the neurotransmitter concentration of the NMDA channel, respectively. b_Mg_ represents the effect of the magnesium block, where we assumed that [Mg^2+^] = 1 mM, as in Tegnér et al. ([2002](http://www.springerlink.com/media/chmntjvqypuhe1lp8fvk/contributions/y/w/w/2/yww25342p87l7752_html/fulltext.html#CR55%23CR55)). We set the constants α_NMDA0_, τ_NMDA0_, α_NMDA_, and τ_NMDA_ to 0.0015 ms^−1^deg^−1^, 2 ms, 0.5 and 100 ms, respectively. Except for α_NMDA0_, the values were taken from Tegnér et al. ([2002](http://www.springerlink.com/media/chmntjvqypuhe1lp8fvk/contributions/y/w/w/2/yww25342p87l7752_html/fulltext.html#CR55%23CR55)). We set the value of α_NMDA0_ by assuming the activation time constant was 30–40 times larger than that of the non-NMDA channel, so that NMDA channels had a relatively slower activation (Koch, [1999](http://www.springerlink.com/media/chmntjvqypuhe1lp8fvk/contributions/y/w/w/2/yww25342p87l7752_html/fulltext.html#CR28%23CR28); McBain and Mayer, [1994](http://www.springerlink.com/media/chmntjvqypuhe1lp8fvk/contributions/y/w/w/2/yww25342p87l7752_html/fulltext.html#CR33%23CR33)).

**The output from the circuit**

The output from this neuron model is defined by 1/(1+exp(−(*V*+15))), which acts as a threshold to convert the membrane voltage into a train of action potentials.

**References -- supplementary material:**

Miura K, Optican LM. Membrane channel properties of premotor excitatory burst neurons may underlie saccade slowing after lesions of omnipause neurons. J Comput Neurosci. 2006 Feb;20(1):25-41.

Huguenard JR, McCormick DA. Simulation of the currents involved in rhythmic oscillations in thalamic relay neurons. J Neurophysiol. 1992 Oct;68(4):1373-83.

Destexhe A, Contreras D, Sejnowski TJ, Steriade M. Modeling the control of reticular thalamic oscillations by neuromodulators. Neuroreport. 1994 Nov 21;5(17):2217-20.

Enderle JD, Engelken EJ. Simulation of oculomotor post-inhibitory rebound burst firing using a Hodgkin-Huxley model of a neuron. Biomed Sci Instrum. 1995;31:53-8.

Traub RD, Wong RK, Miles R, Michelson H A model of a CA3 hippocampal pyramidal neuron incorporating voltage-clamp data on intrinsic conductances. J. Neurophysiol. 1991; 66: 635–650.

Koch C (1999) Biophysics of Computation. Oxford University Press, Inc., New York, NY.

McBain CJ, Mayer ML (1994) N-methyl-D-aspartic acid receptor structure and function. Physiol. Rev. 74: 723–760.

Tegnér J, Compte A, Wang XJ (2002) The dynamical stability of reverberatory neural circuits. Biol. Cybern. 87: 471–481.

Johnson JW, Ascher P (1987) Glycine potentiates the NMDA response in cultured mouse brain neurons. Nature 325: 529–531.

Johnson JW, Ascher P (1992) Equilibrium and kinetic study of glycine action on the N-methyl-D-aspartate receptor in cultured mouse brain neurons. J. Physiol. 455: 339–365.

Thomson AM, Walker VE, Flynn DM (1989) Glycine enhances NMDA-receptor mediated synaptic potentials in neocortical slices. Nature 338: 422–424.

Supplementary figure 1: Schematic presentation of the simulated model.

Supplementary figure 2: Electrophysiological properties of simulated I_h_.
