# Supplementary material for: Hypothetical membrane mechanisms in essential tremor
Source: J Transl Med. 2008 Nov 6;6:68. doi: 10.1186/1479-5876-6-68 (PMC2613385; doi:10.1186/1479-5876-6-68)
Supplement: Additional file 2 — Membrane based model for essential based tremor [file 1479-5876-6-68-S2.pdf]

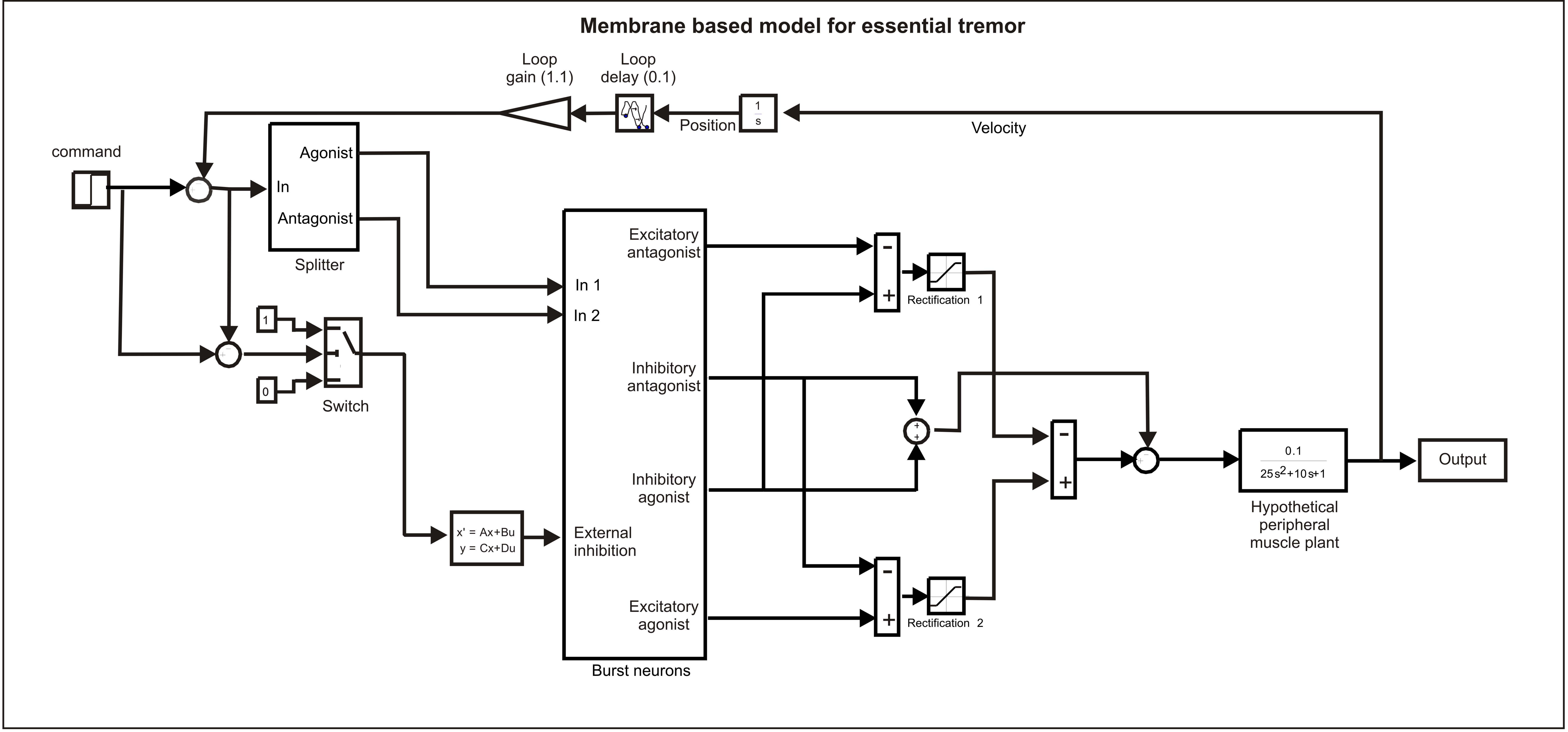

Membrane based model for essential tremor
Loop gain (1.1)
Loop delay (0.1)
1/s
Position
Velocity
command
Agonist
In
Antagonist
Splitter
1
0
Switch
x' = Ax+Bu
y = Cx+Du
Excitatory antagonist
In 1
In 2
Inhibitory antagonist
Inhibitory agonist
External inhibition
Excitatory agonist
Burst neurons
Rectification 1
Rectification 2
0.1
25s2+10s+1
Hypothetical peripheral muscle plant
Output

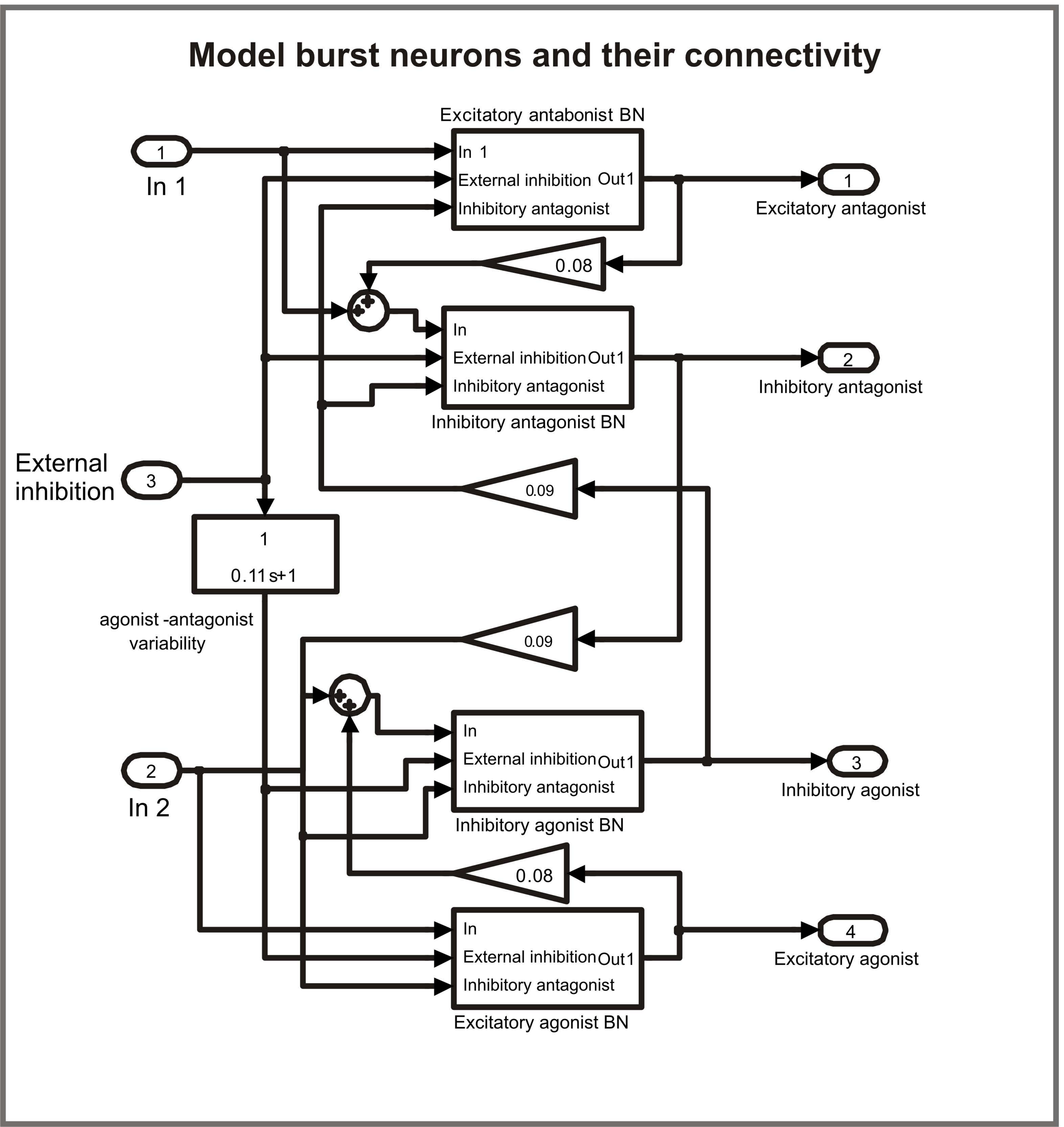

Model burst neurons and their connectivity
Excitatory antabonist BN
In 1
External inhibition
Inhibitory antagonist
Out1
Excitatory antagonist
0.08
In
Inhibitory antagonist BN
Inhibitory antagonist
0.09
External inhibition
1
0.11s+1
agonist -antagonist variability
Inhibitory agonist BN
Inhibitory agonist
In 2
Excitatory agonist BN
Excitatory agonist

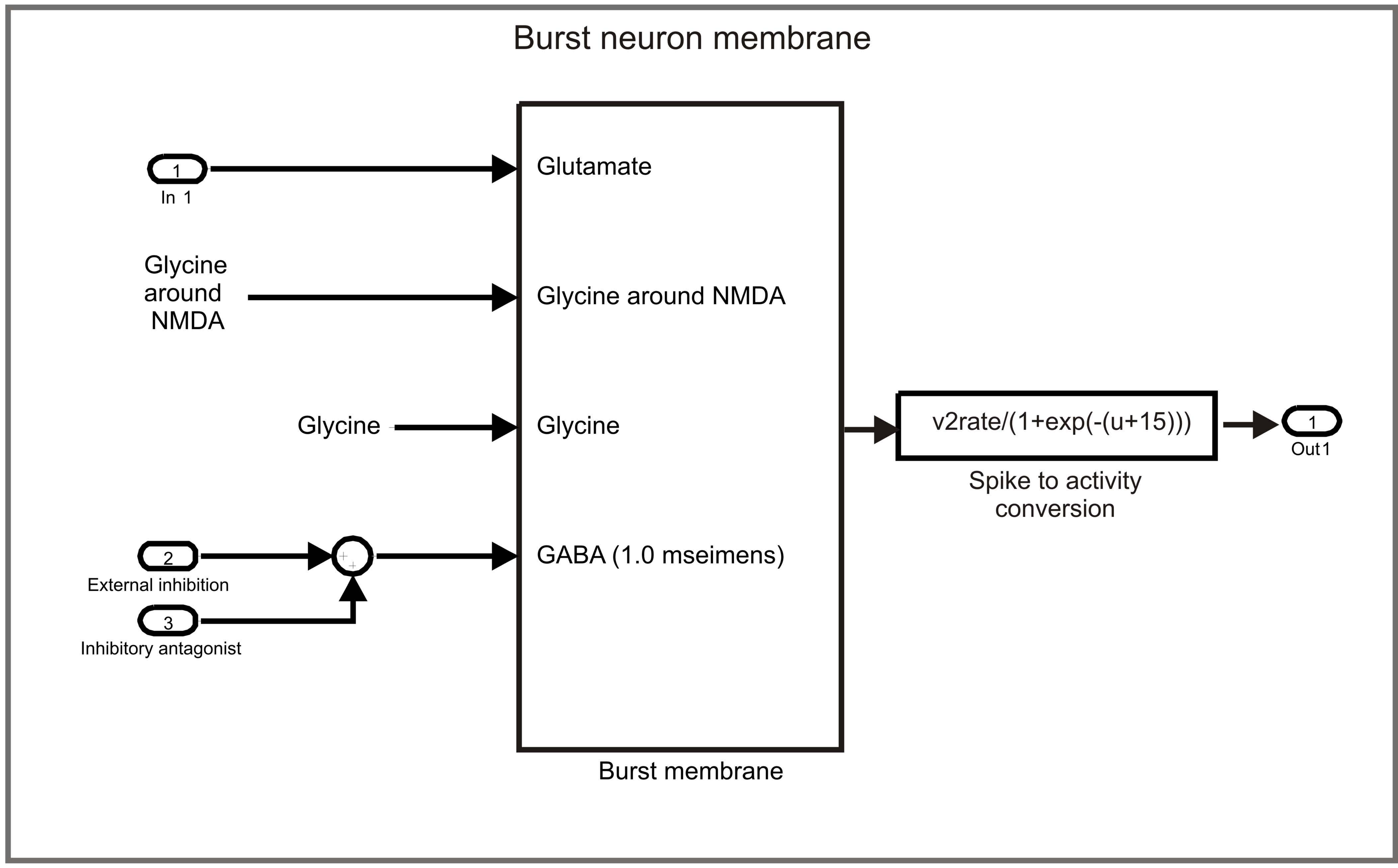

Burst neuron membrane
In 1
Glutamate
Glycine around NMDA
Glycine
GABA (1.0 mseimens)
External inhibition
Inhibitory antagonist
Burst membrane
v2rate/(1+exp(-(u+15)))
Spike to activity conversion
Out1
