# Supplementary figures and images for: Hypothetical membrane mechanisms in essential tremor
Source: J Transl Med. 2008 Nov 6;6:68. doi: 10.1186/1479-5876-6-68 (PMC2613385; doi:10.1186/1479-5876-6-68)

# Voltage dependences of H-current

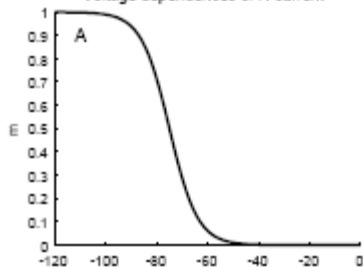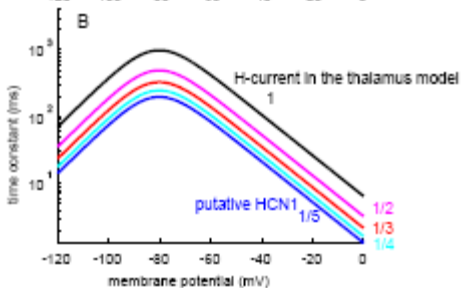

Supplement: Additional file 3. Voltage dependences of H-current [file 1479-5876-6-68-S3.pdf]
